# Genome-Wide Identification and Mapping of NBS-Encoding Resistance Genes in *Solanum tuberosum* Group Phureja

**DOI:** 10.1371/journal.pone.0034775

**Published:** 2012-04-06

**Authors:** Roberto Lozano, Olga Ponce, Manuel Ramirez, Nelly Mostajo, Gisella Orjeda

**Affiliations:** Genomics Research Unit, Faculty of Sciences and Philosophy, Universidad Peruana Cayetano Heredia, Lima, Peru; University of Sydney, Australia

## Abstract

The majority of disease resistance (R) genes identified to date in plants encode a nucleotide-binding site (NBS) and leucine-rich repeat (LRR) domain containing protein. Additional domains such as coiled-coil (CC) and TOLL/interleukin-1 receptor (TIR) domains can also be present. In the recently sequenced *Solanum tuberosum* group phureja genome we used HMM models and manual curation to annotate 435 NBS-encoding R gene homologs and 142 NBS-derived genes that lack the NBS domain. Highly similar homologs for most previously documented *Solanaceae* R genes were identified. A surprising ∼41% (179) of the 435 NBS-encoding genes are pseudogenes primarily caused by premature stop codons or frameshift mutations. Alignment of 81.80% of the 577 homologs to *S. tuberosum* group phureja pseudomolecules revealed non-random distribution of the R-genes; 362 of 470 genes were found in high density clusters on 11 chromosomes.

## Introduction

Plants have developed different strategies to protect themselves from pathogens. Innate resistance in plants can trigger a powerful set of inducible defense responses. One of the most studied mechanisms of defense is mediated by the disease resistance proteins that function in the recognition of pathogen effectors.

Numerous R genes have been cloned from a wide range of angiosperms [1], [2].The most predominant disease resistance genes cloned to date, the NBS-LRR resistance genes, encode proteins containing nucleotide binding (NBS) sites and leucine-rich (LRR) repeat domains. They can, however, also contain additional domains in their amino- and carboxy-terminal domains [3].

The NBS domain was first described as a region spanning 300 amino acids containing several motifs that are strictly ordered [4], [5].This domain is present in an array of plant and animal proteins. In plants, the NBS region is responsible for the binding and hydrolysis of ATP and GTP. Activation of R genes results in cell death through the onset of the hypersensitive response (HR) [6]–[8].

Resistance genes encoding NBS domains are divided into two major groups in plants. These groups are defined by the presence of two domains in the amino-terminal domain, the TOLL/interleukin-1 receptor (TIR) and the coiled-coil (CC) motif [3], [9]. The CC-NBS-LRR (CNL) and the TIR-NBS-LRR (TNL) genes cluster separately in phylogenetic trees [10]–[12]. Both groups are involved in pathogen recognition yet differ in both, amino acid sequences and in their signaling pathway [13].

With access to the full genome sequence, NBS-encoding resistance genes have been annotated in many monocot and dicot species including *Arabidopsis* [9], [14], rice [12], [15], *Medicago truncatula* [11], Poplar [16], grape [5], sorghum [17], *Lotus japonica* [18], *Brassica rapa* [10] and papaya [19]. In these studies, NBS-LRR encoding genes appear as a highly duplicated, evolutionary diverse and clustered gene family [20]. The average percentage of NBS-LRR among all the genes encoded in each organism ranged between 0.6% and 1.76% [19], with *Carica papaya* as the exception, encoding only 54 NBS-LRR proteins, representing 0.2% of its total genes.

The genome sequence of *S. tuberosum* group phureja DM1-3 516 R44 genotype (hereafter referred to as DM), was recently published [21]. In it the ∼740 Mb genome and 39,000 gene complement were described. Included in the analysis and annotation of the potato genome were a rich set of whole transcriptome sequence (PGSC 2011, Massa et al. 2011) and the anchoring of 86% of the genome to the genetic map. Collectively, the availability of the potato genome sequence, annotation, and anchored sequence map permit an in-depth analysis of NBS-LRR genes in this species.

Annotation of disease resistance genes in potato, including positioning them on an anchored sequence map, will permit comparison of NBS-LRR proteins with historical resistance maps [22] and insight into the relationship between R-genes and resistance QTLs. In this study, we identified 435 NBS-encoding genes in the *Solanum tuberosum* group phureja genome (DM1-3 516 R44 genotype). Characterization of these genes included annotation of functional domains, physical position within the genome, congruence with previously reported resistance genes and phylogenetic analyses to investigate their evolutionary relationship. We also identified pseudogenes and partial genes. These analyses provide a robust database of R-gene in potato that will facilitate disease resistance breeding in this important crop.

## Materials and Methods

### Potato genome sequence and annotation resources

Annotated genes (39,031) from the PGSC whole genome annotation of DM assembly were used [21] (PGSC_DM_v3_superscaffolds.fasta.zip; http://potatogenomics.plantbiology.msu.edu/index.html). Whole transcriptome sequence data, RNA-seq, was obtained from the PGSC [23].

### Identification of predicted genes that encode NBS domains

Predicted proteins from DM genome were screened using HMMER V.3 [24] using the raw Hidden Markov Model (HMM) corresponding to the Pfam NBS (NB-ARC) family (UD. PF00931;http://pfam.sanger.ac.uk/). The analysis using the raw NBS domain HMM resulted in 850 candidates. From these, a high quality protein set (<1E -60) was aligned using CLUSTAL W [25] and used to construct a potato-specific NBS HMM using the module “hmmbuild”. With this new potato-specific model, 983 NBS-candidate proteins were identified in total (threshold <1E -2). From the 983 candidate proteins only 435 proteins were selected as NBS resistance candidate genes (Figure S1). This reduction was, in a big portion, due to the similarity between the NBS domains and the Protein Kinase family. Most of the proteins with lower e-values belong to this family and have no relationship to NBS-resistance genes so they were excluded from further analysis.

### Analysis of NBS-associated conserved domains

NBS-encoding resistance genes are often associated with other domains such as TIR and CC in the N-terminal region or a variable number of LRR on the carboxy-terminal region. To detect TIR and LRR domains, Pfam HMM searches were performed. The raw TIR HMM (PF01582) and LRR1 HMM (PF00560) were downloaded from the Pfam database (http://pfam.sanger.ac.uk/) and searched against the final set of 435 NBS-encoding proteins using HMMER V3. Both TIR and LRR domains were validated using NCBI conserved domains and Multiple Expectation Maximization for Motif Elicitation (MEME) [26]. As was previously reported [17], Pfam analysis could not identify the CC motif in the N-terminal region and CC domains were identified using MARCOIL [27] program with a threshold probability of 90 [10] and validated using PAIRCOIL2 [28] with a P score cut-off of 0.025 [19] (Figure S1).

### Resistance-like genes near NBS coding cluster genes

There are some NBS derived resistance genes that cannot be detected by HMM because they lack the NBS domain or have a partial one. To identify such resistance genes near NBS resistance clusters we used a manual method. First, the scaffolds with NBS genes were ordered according to their position inside the chromosome using the pseudomolecules (version 2.1). Then, for each scaffold with NBS, the ORFs were ordered according to their location within the scaffold and the NBS genes were tagged. All the scaffolds with only one NBS gene, or none, were taken out of the analysis. In scaffolds where we found two NBS, we extracted and individually analyzed ORFs from a 100 kb flanking window; if we found three or more NBS genes we increased the window to 200 kb. Each extracted ORF was blasted against the non-redundant protein sequences of NCBI, and selected according to its homology to pathogen stress response or defense genes.

### Alignment and phylogenetic analysis of NBS domains

NBS-containing genes are known to be subdivided in two groups: CC-NBS-LRR (CNL), and TIR-NBS-LRR (TNL). To perform phylogenetic analyses, all 435 NBS-containing proteins were trimmed to extract the NBS domain as revealed by MEME (starting with the p-loop motif). These sequences were aligned using ClustalW [25] with default parameters; the resulting alignment was manually curated using Jalview [29] to remove regions of poor alignment at the end. A phylogenetic tree was constructed using the neighbor-joining method [30] in MEGA 4 [31] with a bootstrap of 500 replicates.

### Anchoring NBS-encoding genes to *S.phureja* genome

The 364 NBS resistance genes (83.7%) and the 106 NBS-derived genes that lack the NBS domain (74.6%) were mapped to their physical position in the genome using the pseudomolecules (version 2.1) provided by the PGSC [21; http://potatogenomics.plantbiology.msu.edu/index.html].

We performed BLASTN between the identified NBS resistance genes and NBS-derived genes against the DM super scaffolds. With the scaffold information the genes were located on the physical map of DM. We used Genomepixelizer [32] to plot the NBS genes into the twelve chromosomes.

### Pseudogene Analysis

A reference R-gene set was built using the Plant Resistance Database [33] and used to find well characterized homologs within our set of potato NBS candidate proteins. The reference set was also used to annotate and classify as pseudogenes those proteins with deletions, insertions or frameshift mutations.

### Experimental evidence of alternative splicing in PGSC0003DMP400023191, a TIR-NBS-LRR-resistance gene

Alternative splicing was shown to be important in the Tobacco Mosaic Virus (TMV) resistance gene [34]. We selected one TMV homolog from the potato genome (PGSC0003DMP400023191) that has the size and structure of a functional TMV to explore if the alternative splicing of this gene is conserved in potato.

Total RNA was extracted from a 14 week old DM plant (leaves stem and roots) using the Tri®Reagent (Sigma). To remove DNA contamination, total RNA was treated with DNA-free™ Kit (Ambion, USA) following the manufacturer's instructions. Reverse transcription was performed with the Transcription First Strand cDNA Synthesis Kit (Roche, USA) using the anchored-oligo(dT)18 primer to generate the first strand cDNA according to the protocol supplied. To analyze the alternative splicing of the TMV resistance gene, multiple primers were designed to amplify the region. Each pair of primers (Red-F: TAATTGTATTCACGGAAGATTATGGA, Red-R: TCAAGAACTACAAGATTTTCATGAGG, Black-F: CTGCTGAAATACAGAATCTCATTGAT, Black-R: ATTTGTTACTTTGTTCAGTGATCTGC, Orange-F: AGAATCTATTGAAGGGCTTGTTCTT, Orange-R: GTCAATATTCACGGGGTCACTC) were screened using re-PCR [34] with both the full assembly genome and the CDS sequence to be sure that each primer pair would amplify just the target region, even after incorporating three mismatches and three gaps per primer. PCR was performed in 50 µL total reaction volume using 5 µL of the first strand cDNAs. Duplicate reactions were performed to validate the presence of each band. PCR products were electrophoresed in a 2.5% agarose gel. Bands were excised and isolated using the Wizard® SV Gel and PCR Clean-Up System (Promega, USA). Each isolated fragment was ligated to the pGEM®-T Easy Vector (Promega, USA). Cloned fragments were sequenced at Macrogen (Seoul, Korea).

## Results

### Identification and classification of NBS genes

A total of 435 non-redundant NBS-encoding R gene candidates were identified in the DM genome (Table 1, Table S1).

**Table 1:** Number of Solanum tuberosum group phureja genes that encode NBS-domains with homology to plant resistance proteins.

Predicted Protein Domains	Code	(PGSC)^a^	Revised^b^
**CC-NBS-LRR**	CNL	60	65
**CC-NBS**	CN	22	24
**NBS-LRR_CC_**	NL_CC_	166	177
**NBS_CC_**	N_CC_	101	104
**Total CNL type**		349	370
**TIR-NBS-LRR**	TNL	35	37
**TIR-NBS**	TN	14	12
**NBS-LRR_TIR_**	NL_TIR_	6	7
**NBS_TIR_**	N_TIR_	4	9
**Total TNL type**		59	65
**Total**		**408**	**435**
**Partial NBS genes** c		N/A	**142**

a Number of R genes identified by the Potato Genome Sequencing Consortium.

b The whole genome annotation and the DNA sequence were screened with proteins that are classified as NBS-LRR in GenBank (3978 sequences).

c Genes that are related to NBS-LRR but has lose the NBS domain or have a very small portion.

It is well known that the NBS domain of resistance genes has some conserved motifs that allow distinguishing between CNL and TNL proteins. This was discovered in *Arabidopsis* where several NBS sub-domains differed between CNL and TNL proteins, giving each NBS domain a specific signature [9].

Analyzing their signature we could classify the 435 NBS-encoding R genes in various groups as shown in table 1 (Figure S2). From the CNL group we found 65 genes with a full NBS resistance structure harboring the three principal domains CC, NBS and LRRs (CNL). In addition, 305 genes belonging to this group lacks a specific motif or domain and were classified in three distinct groups; CN (24), NL (177) and N (104) (Figure S2).

The 65 TNL resistance proteins were also distributed as follows; 37 TNL, 12 TN, 7 NL and 9 N (Figure S2).

A majority of the disease-resistance reference genes previously reported in Solanaceae species had high sequence identity with our set of candidate R genes including Hero[35], R1 [36], BS4 [37], Rpi-Blb2 [38], and Gpa2 [39], of 13 reported genes examined all were found in our set (Table S2). When the total 435 NBS resistance proteins were compared to Uniref, 230 had homology to known-function disease resistance proteins. This shows, at least to some degree, that our analysis was deep enough to identify well characterized genes.

Analysis of NBS resistance genes in *C. papaya* [19], showed various types of proteins with similarity to resistance genes near NBS-domain resistance clusters. These genes have in many cases shown homology to resistance genes different from NBS. To explore this observation in DM potato, proteins located adjacent to NBS clusters were analyzed and several proteins (191) located near NBS clusters were identified with homology to resistance genes (biotic and abiotic) (Table S3). From those, 142 have homology to members of the NBS family but do not have (106) or contain only a small portion of the NBS domain (36) (Figure S2), consistent with their lack of detection via HMM analysis. These genes were not considered in the main list of NBS-coding genes (Table S1).

### Phylogeny construction

The amino acid sequence of the NBS domain of each predicted NBS resistance protein was extracted and used to perform a phylogenetic analysis. Proteins with an incomplete NBS domain were not included (Figure S3). For comparative purposes, we included well-characterized cloned resistance genes from *Arabidopsis thaliana*, *Oryza sativa*, *Solanum lycopersicum*, and *Solanum tuberosum* (Table S4, red in Figure S3). A total of 224 NBS domains were aligned and three different large clades were evident.

One of these large clades, (CC(I)), includes most of the CC-type proteins, including highly related homologs of Late blight resistance protein BLB2, HERO, PRF or BS2. Most of the known-function CC-type proteins are contained within the CC(I) clade. Most reference proteins are positioned into clusters with related homologs from potato; however there is a large clade of known R-genes from grasses that miss a potato homolog. This cluster groups proteins such as NBS4-Pi, PIZ-T, PI-TA, PI36 (*O. sativa* sp indica), LR10 (*Triticum aestivum*) and MLA6-10-12 (*Hordeum vulgare*), which was expected since NBS-LRR proteins from grasses have some unique characteristics [12], [15].

The CC(II) clade contains only 25 NBS resistance proteins with the majority of them with homology only to putative or hypothetical proteins. Only three reference genes were present within this clade, RPS5 and RPS2 from *A. thaliana* (CNL-B, [9]), and a resistance protein candidate RGC2B from *Lactuca sativa*. This high degree of separation between two CNL groups has not been observed in other studies.

The third clade groups all TNL proteins and shows clear separation between these groups and the CNL proteins, consistent with previous studies in different species [9]–[11], [19]. We can find in these clade homologs to RPP1-4-5, (*Arabidopsis thaliana*) and Bacterial spot disease resistance proteins (*Solanum lycopersicum*) among others, however some proteins like RPS4 (*Arabidopsis thaliana*) cluster separately from the potato resistance gene candidates.

**Figure 1:**
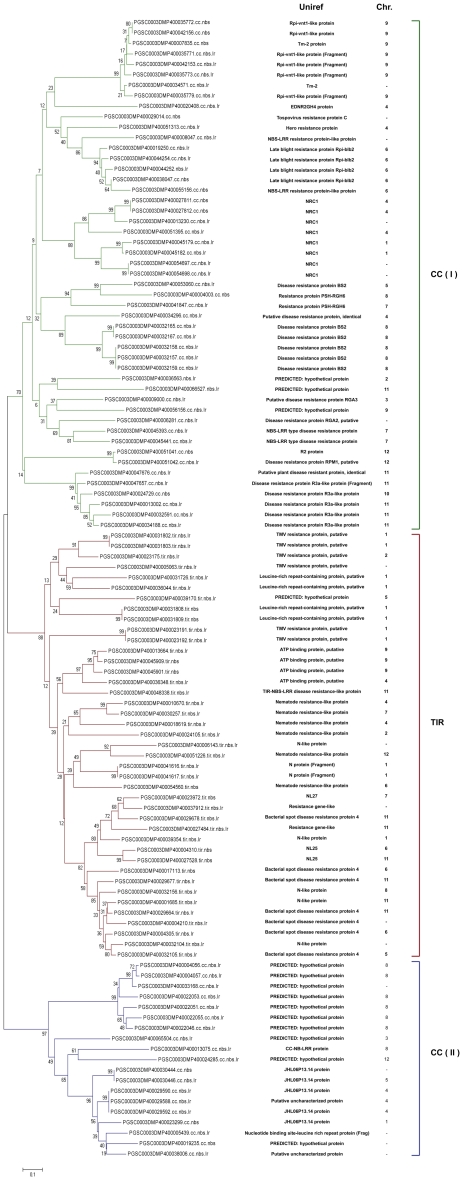
Phylogenetic construction of the NBS-LRR proteins in *Solanum tuberosum* group *phureja*. The neighbor-joining tree was constructed using the sequences of 110 NBS-containing proteins using MEGA4 software [31]. Sequences were trimmed to extract just the NBS domain. Bootstrap values are indicated on the branches. Each protein is encoded by its full PGSC code followed by its type (CNL, TNL, TN and so on); green, blue and red branches correspond to CC(I), CC(II) and TIR clades respectively. Functional annotation and chromosome location for each protein are positioned in the right margin.

For ease of visualization, we selected 110 potato representative proteins to construct another tree without the known R genes from other species to show the relationship among the potato NBS resistance genes (Figure 1). This new tree shows similar results, three major clades: CC (I), CC (II) and TIR. The clear separation between CC (I) and CC (II) clades confirms that this separation was not an artifact of the last tree mediated by the known genes that were added.

The cluster nature of the resistance genes is well represented in this tree. In the CC(I) group we found a cluster of homologs of Rpi-vnt1 (chromosome 9), Rpi-blb2 (chromosome 6), NRC1 (chromosomes 4 and 1) and R3 (chromosome 11). There is one notable cluster of the CC(II) type composed of proteins with homology to a not-yet-characterized and hypothetical protein according to Uniref positioned on chromosome 8.

There was no problematic grouping: all TNL and TN types grouped into the TIR clade; and those CNL and CN grouped together into CC(I) or CC(II) clades (Figure 1). N/NLs were dispersed among both groups (Figure S3); as previously mentioned [16], this behavior indicates a diverse rather than monophyletic origin of this group of proteins. Proteins belonging to this group (N/NL) must be originated by a domain loss (that in most cases leads to the creation of pseudogenes), and in a very low frequency due to errors in our analysis or in the annotation. Errors in the start codon position or an exon skipped during the annotation would lead to a misidentification of a domain in the N terminal region.

Due to the striking differences between CC groups, we selected all the CC(II) proteins and a representative number of CC(I) proteins and used MEME and local alignments, using ClustalW to examine the differences between these groups of proteins, not only with respect to the NBS domain but also with respect to the N- and Carboxy-terminal regions.

**Figure 2:**
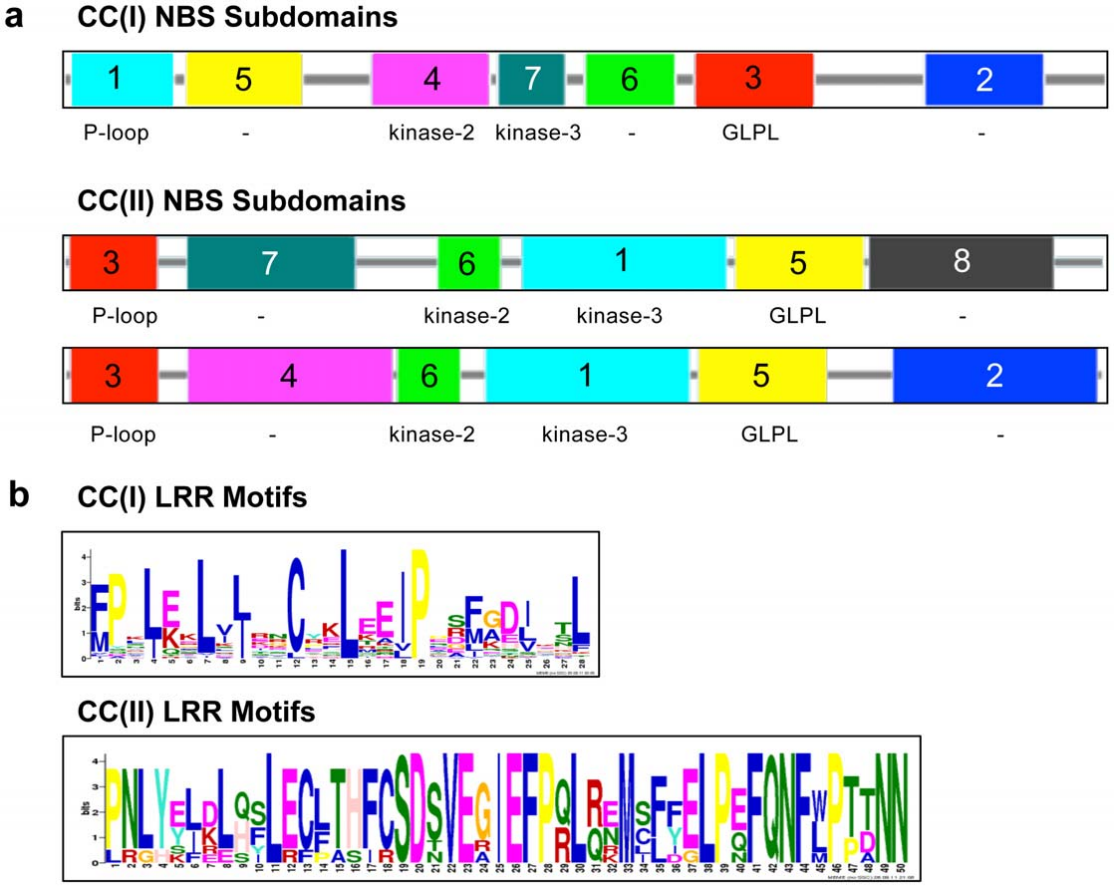
MEME analysis of NBS and LRR regions between CC(I) and CC(II) proteins. (**a**) NBS domain analysis. Different color boxes represent different subdomains. CC(I) and CC(II) were analyzed separately. Even though CC(II) has two different configurations, (subdomains 7-4 and 8-2) they are clustered together due to strong similarities on principal subdomains (P-loop, kinases and GLPL. Figure S5). (**b**) Predominant LRR motifs are also different between these two groups; CC(I) proteins have smaller and more abundant LRR. Different color letters represent amino acid belonging to different families.

In general, the CC(I) and CC(II) NBS regions share the main subdomains, P-loop, kinase-2, kinase-3 and the GLPL, but also have at least two regions with high divergence. The first region is found between the P-loop and the kinase-2, and the second one is near the GLPL subdomain (Figure 2). Members of the CC(II), despite having two very different domains in the areas previously mentioned, still cluster together in the phylogenetic analysis due to the conservation of the principal subdomains. The difference between groups CC(I) and CC(II) is notable if we align these two subgroups (visualized with Jalview. Figure S4; ClustalW raw output, Table S5). If we analyze the CC(I) and CC(II) proteins in depth, we observe clear differences in the CC and carboxy terminal regions. Most of the CC(II) proteins have an additional domain at the N terminal domain that is not present in the CC(I) group (Figure S5). Also, the LRR motifs are quite different between these groups, in general CC(I) has a greater number of a smaller LRR motif (Figure 2; Figure S5).

### Genomic Distribution

**Figure 3:**
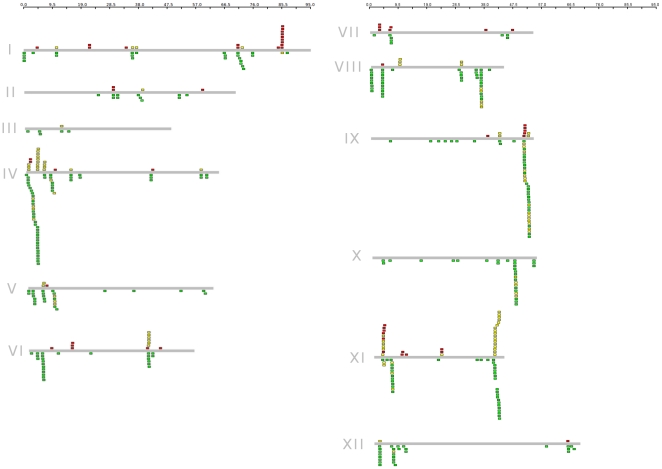
Distribution of *Solanum tuberosum* group *phureja* sequences that are predicted to encode NBS resistance proteins. Gray bars represent all 12 linkage groups in potato. Boxes across each bar designate the location of each gene. Color code: CNL (green), TNL (red) or a partial NBS gene (yellow). Distance in Megabases is shown at the top of each column.

Using the current version of the physical map (PGSC_DM_v3_2.1.10_pseudomolecule_AGP.xlsx) 72 (16%) DM NBS-encoding genes are on unanchored scaffolds. Chromosome 4 has the highest number of R-genes (55, 12.6% of mapped genes) and the most underrepresented chromosome is chromosome 3, with just 5 genes with none of genes clustered (Table S6). It is evident that the distribution of these genes among the majority of chromosomes is not even (Figure 3). This unequal distribution of NBS-encoding genes is not novel in the plant genomes [5], [9]–[11], [15], [16], [19]. The clustered nature of these genes is thought to facilitate the evolution of R genes through sequence exchange via recombinational mispairing [40].

There were more CNL tandem duplicates than TNL, and the CNL clusters are much larger than the TNL clusters. This may be explained in part due to the predominance of CNL genes in potato (85%). These clusters are in most cases homogeneous (highly similar to other NBS in the same genomic cluster). However, there are several cases where TNL genes are mixed with CNL genes in close super clusters. Truncated R genes lacking large portions of the NBS domain or the LRR region are often associated with full R-genes (Figure 3 in yellow; Table S3).

TNL genes tend to form small clusters of up to 9 genes. Only 12 TNL proteins (19.6% of anchored TNL genes) are not grouped (singletons); most members of this family are located within clusters with only two or three genes; 26 (42.6%) and 23 (37.8%) belong to clusters with more than four members. One exception is a cluster on chromosome 1 that has 9 of these genes, with similarity to the TMV resistance protein (Figure 3). The other clustered TNL genes are similar to Bacterial spot disease resistance proteins, NL25 and a putative disease resistance protein. We found out that there is presence of TIR genes among the entire genome except for chromosomes 3 and 10. However, in chromosomes 8 and 12 we could only find one TIR gene each, and neither was annotated as a pseudogene, suggesting that genes that are not clustered together could be more stable. However, of the 9 unclustered TNL, 4 are pseudogenes due in most cases by premature stop codons.

Interestingly, although a large number of TNL genes do not occur physically in clusters, most of them are located near CC clusters. PGSC0003DMP400032156, for example, is a TNL gene with homology to N protein that is positioned at 3.71 Mb in chromosome 8 (Figure 3) near a CC cluster (starting at 3.73 Mb) of BS2 homologs. Few examples show TNL genes “alone” in the genome. One of these examples, however, is present in chromosome 7 where two genes, PGSC0003DMP400023972 (38.35 Mb), and PGSC0003DMP400030257 (47.13 Mb), can be found “alone” (>3 Mb from any NBS-gene). PGSC0003DMP400023972 has the structure of a full NBS gene with homology to a nematode resistance protein; however PGSC0003DMP400030257 has a frameshift mutation and lacks the LRR domain. It is interesting to note that the five remaining TNL genes in these chromosomes are also tagged as pseudogenes. Another example of TNL-alone genes is PGSC0003DMP400017113 on chromosome 6, encoding a full Bacterial spot disease resistance protein 4 homolog.

On the other hand, CC proteins group in large clusters which form super clusters. For example, at ∼50 Mb on chromosome 9 a group of 45 proteins occur in a 2 Mb region. This super cluster is an interesting example as it comprises four clusters (three CC clusters and one TIR cluster (four members). More common examples are super clusters that group only CC proteins such as the one starting at ∼41 MB in chromosome 11 (Figure 3).

### Pseudogenes

When the 435 NBS resistance genes were closely analyzed, a total of 179 pseudogenes were identified across the twelve linkage groups of potato. Most of these pseudogenes have very strong identity with another full NBS protein but their sequence is truncated by either a premature stop codon or a frameshift mutation (Table S7). We have also observed that some of these pseudogenes resemble partial genes and appear to be the product of deletions. More rare events are reported, such as some cases of pseudogenization by transposition, exon skipping, and a single case where the scaffold ends leading to a truncated gene (Table S7). This very high number of pseudogenes, however, may be an underestimation as the pseudogenes identified have at least a partial NBS domain; other pseudogenes that might have lost the whole NBS domain are not taken into consideration as they were not included in this analysis (Table S3).

The distribution of pseudogenes among the chromosomes is described in Table 2. Chromosome 3 groups only five NBS-coding genes, and only one of them (20%) is annotated as pseudogene; chromosome 1 has 40 genes, and only 12 (30%) are classified as pseudogenes. In contrast, chromosomes 6 and 9 have higher ratios of pseudogenization; 54.84% and 57.89% respectively. The number of pseudogenes also varies between groups. Counts of CNL and TNL-like pseudogenes are 156 and 23 respectively, which was expected, since 85% of the total NBS-genes are CNL group while only 15% are TNL-like.

**Table 2:** Distribution of pseudogenes across the chromosomes and by domain group.

Chromosome	total genes	# pseudogenes	%
**chr1**	40	12	30,00
**chr2**	14	4	28,57
**chr3**	5	1	20,00
**chr4**	55	24	43,64
**chr5**	24	10	41,67
**chr6**	31	17	54,84
**chr7**	15	6	40,00
**chr8**	42	14	33,33
**chr9**	38	22	57,89
**chr10**	25	8	32,00
**chr11**	47	18	38,30
**chr12**	27	11	40,74
-	72	32	44,44
**Total**	**435**	**179**	41,15

Type	total genes	# pseudogenes	%
**CC**	370	156	42,16
**TIR**	65	23	35,38
**Total**	**435**	**179**	41,15

**Figure 4:**
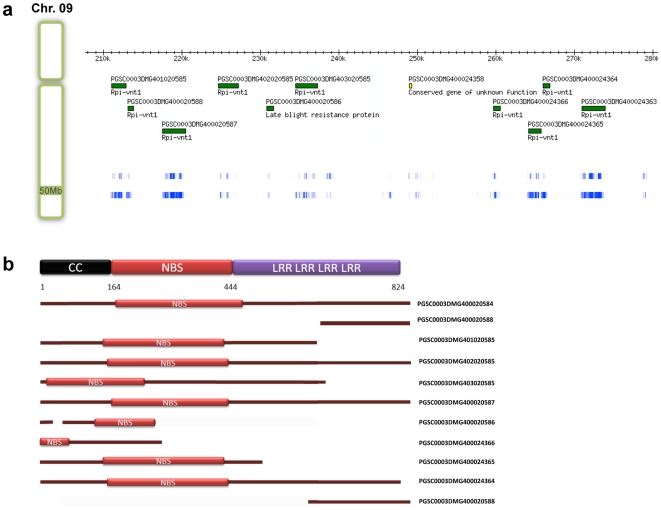
Rpi-vnt1 cluster structure. (**a**) This cluster is positioned at ∼50 Mb in chromosome 9. The exact position of each gene-model is represented by a green rectangle. For reference we include the relative expression of each gene as obtained by RNAseq of leaves in normal condition and leaves infected with *Phytophthora infestans*. As these genes encode homologs to an Rpi protein we expected that the expression would increase during infection. (**b**) Structure of the proteins encoded by the genes in (**a**). At the top we can see a representation of the reference Rpi-vnt1 gene. All the genes presented are encoded within the cluster except for PGSC0003DMG400020584 that is placed at position 33 kb of the same scaffold. These proteins share a very high homology with the reference protein.

As observed in other plants [11], most of the potato NBS pseudogenes (>90%) are within 100 kb from another NBS gene. In fact, the typical clustered nature of these proteins can be observed in position 50 Mb of chromosome IX. The Rpi-gene cluster located in the scaffold PGSCDMB0000339 and we identified nine pseudogenes within 100 kb, plus another pseudogene 200 kb upstream from this cluster. As some of these genes may remain functional, some of them have become pseudogenes and their breakpoints are shown (Figure 4).

As shown previously (Table S7), some of the pseudogenes have evidence of expression. Expression of pseudogenes has been observed in other organisms [11], [15], [18]. In mouse there was a confirmation that an expressed pseudogene was involved in the stability of its mRNA functional homologue [41], but this behavior has not been observed in plants yet.

### Resistance-like genes near NBS coding clusters genes

Regarding the function of the genes near NBS clusters, over 64% are homologs to resistance genes, the rest have similarity to proteins implicated in stress, abiotic and biotic responses. For example, PGSC0003DMP400004954 has significant similarity to Ferroportin, a protein necessary for iron and cobalt homeostasis [42], PGSC0003DMP400005064 is homolog to Nicotianamine synthase, which increases nickel tolerance and iron use [43], PGSC0003DMP400005224 and PGSC0003DMP400062326 have significant similarity to dehydration-responsive element binding protein, PGSC0003DMP400005210 to AP2/ERF domain-containing transcription factor, and together are induced in roots from *Capsicum annuum* by dehydration, high salinity and mechanical wounding [44], PGSC0003DMP400051306 is homolog to Oligouridylate binding protein, a gene early expressed during *Coffea arabica* infection by rust fungus [45].

### Alternative splicing

**Figure 5:**
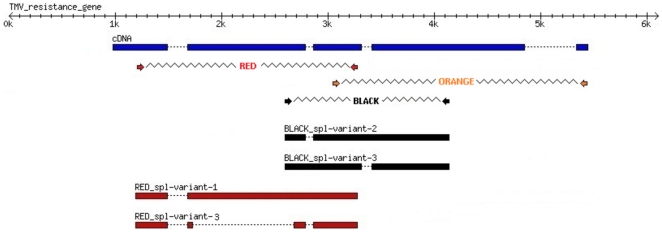
Alternative splicing of a TIR-NBS-LRR resistance gene. Three different primer pairs (orange, red and black) were used to explore alternative splicing in the gene PGSC0003DMP400023191 with homology to a TMV resistance gene. cDNA from 14 week old DM plants was amplified and products with different sizes were cloned and sequenced. cDNA sequence was then blasted to the resistance gene DNA sequence at scaffold PGSC0003DMB000000105. Dotted lines represent introns.

PGSC0003DMP400023191, a TMV homolog was tested to explore if the alternative splicing of this gene was conserved in potato. Three pairs of primers were used to amplify the cDNA (Figure 5).

Red primers amplification identified two transcripts that differ from their expected structure, one transcript showed an intron retention (intron 2) resulting in a 1,5 kb fragment while the other showed the loss of a big portion of the second exon (that encodes the NBS domain). Black primer also showed two splicing variants, both were “intron retention” type (intron 2 and 3 respectively). Amplification with the Orange primer resulted in 3 bands of different sizes than the expected; however these bands could not be cloned.

## Discussion

Cultivated potato, *Solanum tuberosum* (2n = 4x = 48) is the world's most important non-grain food crop. It belongs to the Solanaceae family that included economically important species such as tomato, pepper, eggplant, and tobacco. Potato is not only important as a fresh market product, but has become of great interest for the French fries, chips and starch processing industries. Potato, however, is susceptible to many diseases and its growth is dependent on a huge amount of pesticides. Given the importance of this crop, improvement of disease resistance is essential, however, classical breeding of an autotetraploid species is a challenge. Moreover, asexual propagation and numerous market limiting traits complicate the task. The availability of the potato genome sequence provides us with the opportunity to identify the complement of NBS-containing R gene homologs as a first step in marker assisted genetic improvement or genomic selection of this vital crop.

NBS-LRR genes are the largest class of disease resistance proteins and have a major effect on the defense of the plant against its pathogens. We have initially found 435 NBS-containing proteins in the potato genome; this number is close to that found in poplar (402, [16]). If we also consider those proteins related to this family that have lost the NBS domain, the number simply gets bigger. The ∼577 NBS members (435) and cluster associated NBS-derived genes lacking the NBS domain (142) represent ∼1.48% of all the proteins predicted in potato. This number is higher compared to rice (∼1%, [15]), *A. thaliana* (∼0.43%, [9]), and *Populustrichocarpa* (∼1%, [16]).This analysis was performed on a double monoploid potato but considering the highly heterozygous behavior and the haplotype diversity observed in the diploid *Solanum tuberosum* RH genotype (PGSC. 2011) it's possible to envisage that a cultivated tetraploid potato may contain thousands of R genes.

The largest class of NBS in *S. phureja* is represented by non-TIR genes. The predominance of TNL or CNL genes through the genome may be determined by the pathogens that infect the plant species throughout its history or may be driven by another evolutionary force that could be related to the success of one or another type of R genes. TNL are the predominant class in *A. thaliana* [9] and *Brassica rapa* [10]; however, in *P. trichocarpa*, as in potato, CNL are the most abundant class [16]. A more drastic separation can be observed in the cereals where TNL genes have not been found; moreover, cereals have CNL genes that have no homologs among the dicot species [15]. This behavior is observed also in potato, where a large group of CNL cereal reference R-gene clusters separate from any CC gene (Figure S3). This evidence supports the idea that the resistance gene arsenal of dicot and cereal species have greatly diverged since their independent evolution [15].

According to Freedman and Baker (2007), R-gene evolution and quantity is dependent on the size and cluster complexity of these genes. The high number of resistance genes identified in this study could be explained mainly by the size of the NBS-clusters. The number of resistance-like genes near NBS coding clusters follows the same principle; the chromosomes with the highest number of these genes have the biggest clusters, for example chr1 and chr11. The low presence of the TIR domain could be explained by the fact that most of the TIR genes are either inside small clusters or out of any clusters.

The clusters identified in this study can be classified in mixed, simple and complex, and the genes near them have shown the same trend. Complex clusters have resistance and stress related-genes with varying domains. This attribute could be explained by intergenic recombination enhanced by R-gene clusters, previously hypothesized by Freedman and Baker (2007). It is therefore expected that these R-related genes keep growing in number, size and complexity in the potato genome, and expand the different functions of these genes in this crop.

Although most of the genes mapped in this study are grouped in clusters (77%) we found some singleton genes. As observed in other studies [11] these singletons have homologs elsewhere in the genome. One hypothesis is that these solitary genes may act as “seeds” establishing the position for new clusters [11]. This idea must be revised as the singletons may be in a more stable genome region just by chance; studying the distribution and dynamics of these singletons from different potato species would allow us to make more inferences on this issue; in fact, a large proportion of CNL singleton genes in the DM genome are pseudogenes.

NBS resistance genes have a very rapid turnover, especially those that are encoded within clusters where large numbers of both new genes and pseudogenes are generated. We have found that in potato there are a very high number of pseudogenes that reaches 41.6% of the total R-genes found in potato. This percentage is very close to that observed in a previous but less profound analysis (39.4%, [21]). This unusual high rate of pseudogenization might parallel the rapid evolution of effector genes found in potato pathogens such as *Phytophthora infestans* [21].

The pseudogene percentage in potato is very high if we compare it to similar analyses made in other plant genomes such as *Arabidopsis thaliana* (8.05%, [9]) or *Medicago truncatula* (14.74%, [11]). On the other hand, in *Lotus japonica* a very high percentage of pseudogenes (39%, [18]) was also found. We believe that the pseudogene rate found in each species may be the product of a criterion bias, among other factors. In the two articles mentioned above, the authors had relatively restrictive criteria for identifying pseudogenes. Establishing parameters for selecting pseudogenes may help to reduce the difference but this may not be the only way to overcome this variability, as there are too many variables to take into consideration.

As pseudogenes could be non-functional genes that are just waiting to be eliminated from the genome, or reservoirs of genetic diversity that could be reached under recombination or gene conversion [13], a new hypothesis of their real function is taking shape. As mentioned before, it was demonstrated in mouse that a pseudogene was capable of regulating the messenger-RNA stability of its homologous coding gene [41]; moreover a recent investigation in tumor cells has demonstrated that expressed pseudogenes can regulate coding gene expression, as they compete for microRNA binding, thus revealing a non-coding function for pseudogenes [46].

It is interesting that of the total 179 pseudogenes, 74 have evidence of being expressed by RNAseq analysis, 42 of those are expressed with a mapping depth higher than 10 FPKM (Fragments per Kb of exon per million reads) and a couple of these are expressed with values higher than 100 FPKM. To our knowledge, this is the first analysis in plants that uses available RNAseq information to analyze pseudogene expression. Expressed NBS-LRR pseudogenes have been reported for *Medicago truncatula* [11], pine [47], and rice [15].

The pseudogenes presented in potato (Tables S7) plus the more evident NBS-truncated proteins found around the NBS-clusters (Table S3 yellow) could act as adaptor molecules, acting as recruiters or merely interacting with the NBS-LRR genes [48]. The products of the pseudogenes would be very similar to those that arose from alternative splicing as in TMV resistance protein (N); it was shown that in the presence of TMV p50 elicitor the resistance protein tends to oligomerize [49] and that in such conditions an N alternative splicing that lacks the LRR region is overexpressed [50]. Taking these facts together, it was hypothesized that oligomerization of alternate N proteins may be crucial for the stability of N and the HR response [40]. The expression of alternatively splicing isoforms derived by a single TMV resistance protein gene was experimentally confirmed in potato (Figure 5).

In this scenario, different isoforms derived from alternative splicing may act in the same way as pseudogenes. As reviewed previously [51] the phenomenon of alternative splicing is best described among TNL genes, as they generally have more exons than CNL genes, which in many cases are intronless, as are RPS2 [52], RPM1 [53], and RPS5 [54]; moreover, no alternative splicing has been detected among any intronless Arabidopsis CNL gene. CNL alternative phenomenon was described only for the Mla locus in barley [55]; however, this family has no homologs among potato NBS genes.

It was tentatively thought that the infrequency of CNL alternative isoforms may be the reason why they form larger clusters than TNL genes in potato. In some CNL clusters, the proteins within this region have strong homology relationships as shown in figure 4, and some of them may be acting in a similar way as the alternative isoforms of N protein described previously. Large clusters of TIR genes have been observed in *Arabidopsis* [9] and *Brassica rapa* [10], indicating that the ability to form alternative isoforms may not be related to the cluster nature of some R-proteins; this observation, however, needs more evidence to be cleared.

It was recently discovered, as mentioned above, that expressed pseudogenes could regulate their coding gene expression by competing for microRNA binding [46]. Following this observation, NBS pseudogenes and truncated NBS genes may be preventing the degradation of their homologous functional R-genes by the local silencing system. It would be interesting to explore miRNAs in the potato genome targeting NBS genes and infer whether there is some kind of particular miRNA-driven regulation of these genes. A more appealing hypothesis would be that this kind of “protection” was originated in the coevolution of pathogens with plants. It is not yet clear, but there is published evidence that some viral siRNAs could be targeting host genes [56] and the expression of pseudogenes or the formation of alternative isoforms may be the plants way of countering the viral strategy to suppress their defense system.

This analysis on the genome-wide distribution of R gene sequences within potato is the first effort to understand the mechanisms of the evolution among resistance genes in the Solanaceae family. It would be of great interest to compare the number, cluster positions, and evolution of closely related organisms. With the upcoming sequencing of the tomato we would be able to compare two organisms with high levels of synteny in their sequences and genome structure that have different life cycles and pathogens. This comparison could shed some light on the dynamics and recent evolution of NBS genes.

## Supporting Information

Figure S1 NBS resistance protein identification workflow.
(PPTX)

Figure S2 NBS resistance genes by its domains.
(PPTX)

Figure S3 Phylogenetic tree including 224 NBS resistances genes found in this study plus reference R genes cloned from different species (in red).
(PPT)

Figure S4 Alignment between CC(I) clade proteins vs. CC(II) clade proteins visualized using JALVIEW.
(PNG)

Figure S5 Differences between the domains of CC(I) and CC(II) proteins visualized with MEME.
(PPTX)

Table S1 List of the 435 NBS resistance proteins.
(XLSX)

Table S2 BLAST results of cloned resistance genes against the NBS resistance proteins found in *Solanum tuberosum* group phureja.
(XLS)

Table S3 List of the 190 proteins with homology to resistances proteins (biotic or abiotic) found near NBS-resistance cluster.
(XLSX)

Table S4 List of Reference R genes included in the phylogenetic tree.
(XLS)

Table S5 Raw alignment of CC(I) clade proteins against CC(II) clade proteins.
(ALN)

Table S6 List of the NBS resistance proteins mapped to its position in the chromosomes.
(XLSX)

Table S7 List of NBS resistance proteins tagged as pseudogenes.
(XLSX)
