# Supplementary material for: Genome-Wide Identification and Mapping of NBS-Encoding Resistance Genes in Solanum tuberosum Group Phureja
Source: PLoS One. 2012 Apr 6;7(4):e34775. doi: 10.1371/journal.pone.0034775 (PMC3321028; doi:10.1371/journal.pone.0034775)
Supplement: Figure S1 — NBS resistance protein identification workflow. (PPTX) [file pone.0034775.s001.pptx]

## Slide 1
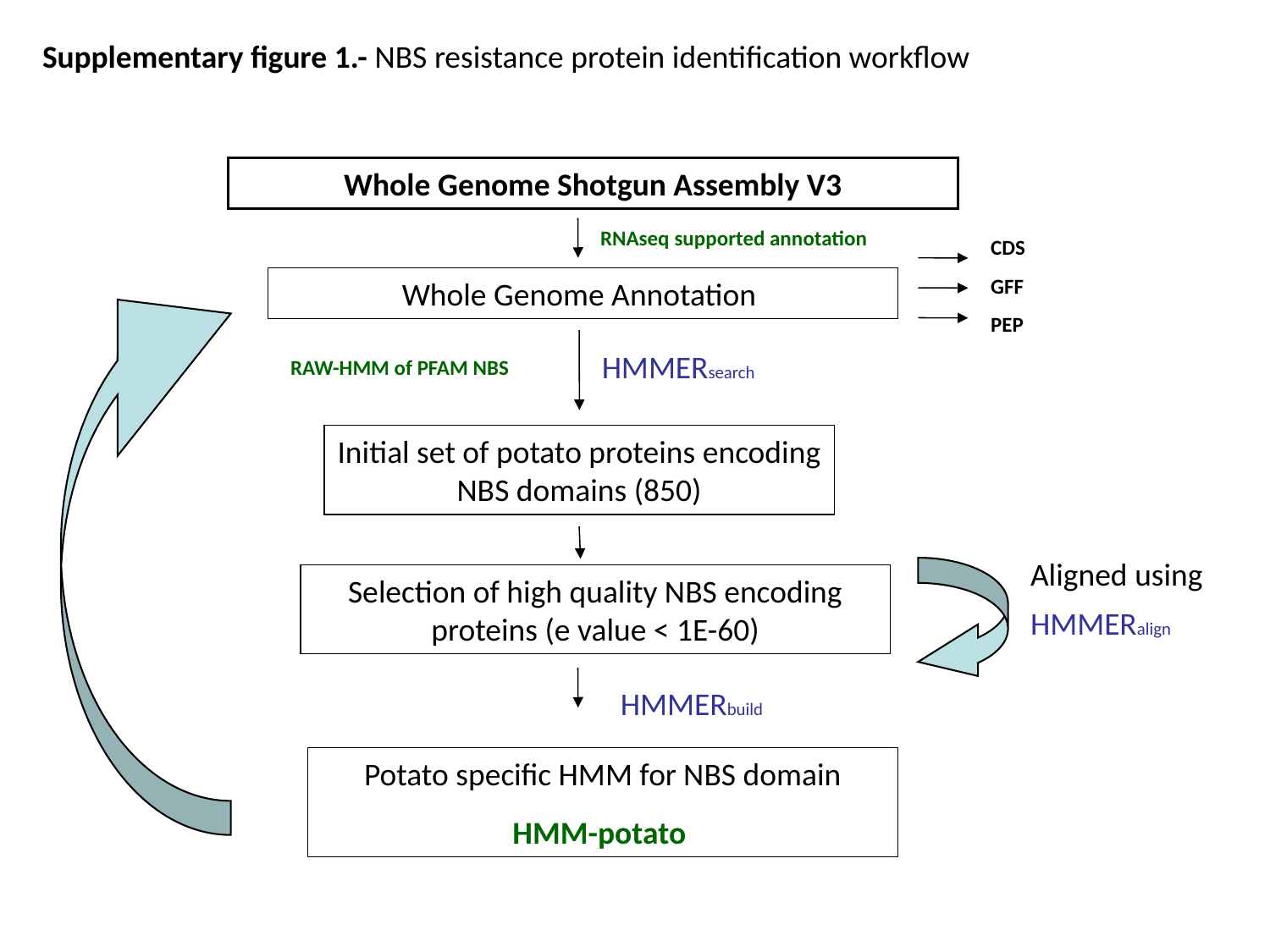

Supplementary figure 1.- NBS resistance protein identification workflow
Whole Genome Shotgun Assembly V3
RNAseq supported annotation
CDS
GFF
PEP
Whole Genome Annotation
HMMERsearch
RAW-HMM of PFAM NBS
Initial set of potato proteins encoding NBS domains (850)
Aligned using
HMMERalign
Selection of high quality NBS encoding proteins (e value < 1E-60)
HMMERbuild
Potato specific HMM for NBS domain
HMM-potato

## Slide 2
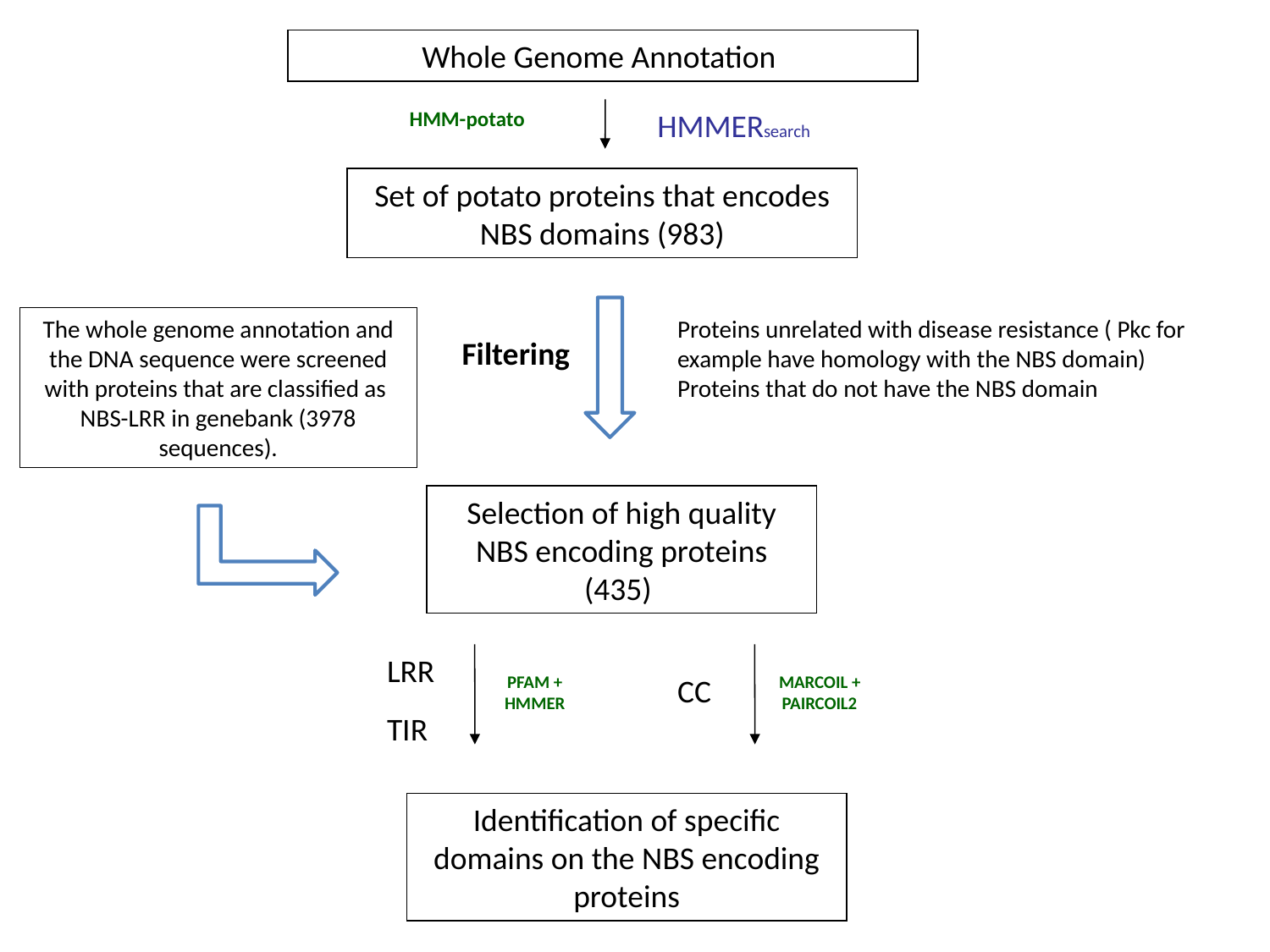

Whole Genome Annotation
HMM-potato
HMMERsearch
Set of potato proteins that encodes NBS domains (983)
The whole genome annotation and the DNA sequence were screened with proteins that are classified as NBS-LRR in genebank (3978 sequences).
Proteins unrelated with disease resistance ( Pkc for example have homology with the NBS domain)
Proteins that do not have the NBS domain
Filtering
Selection of high quality NBS encoding proteins (435)
LRR
TIR
PFAM + HMMER
CC
MARCOIL + PAIRCOIL2
Identification of specific domains on the NBS encoding proteins

## Slide 3
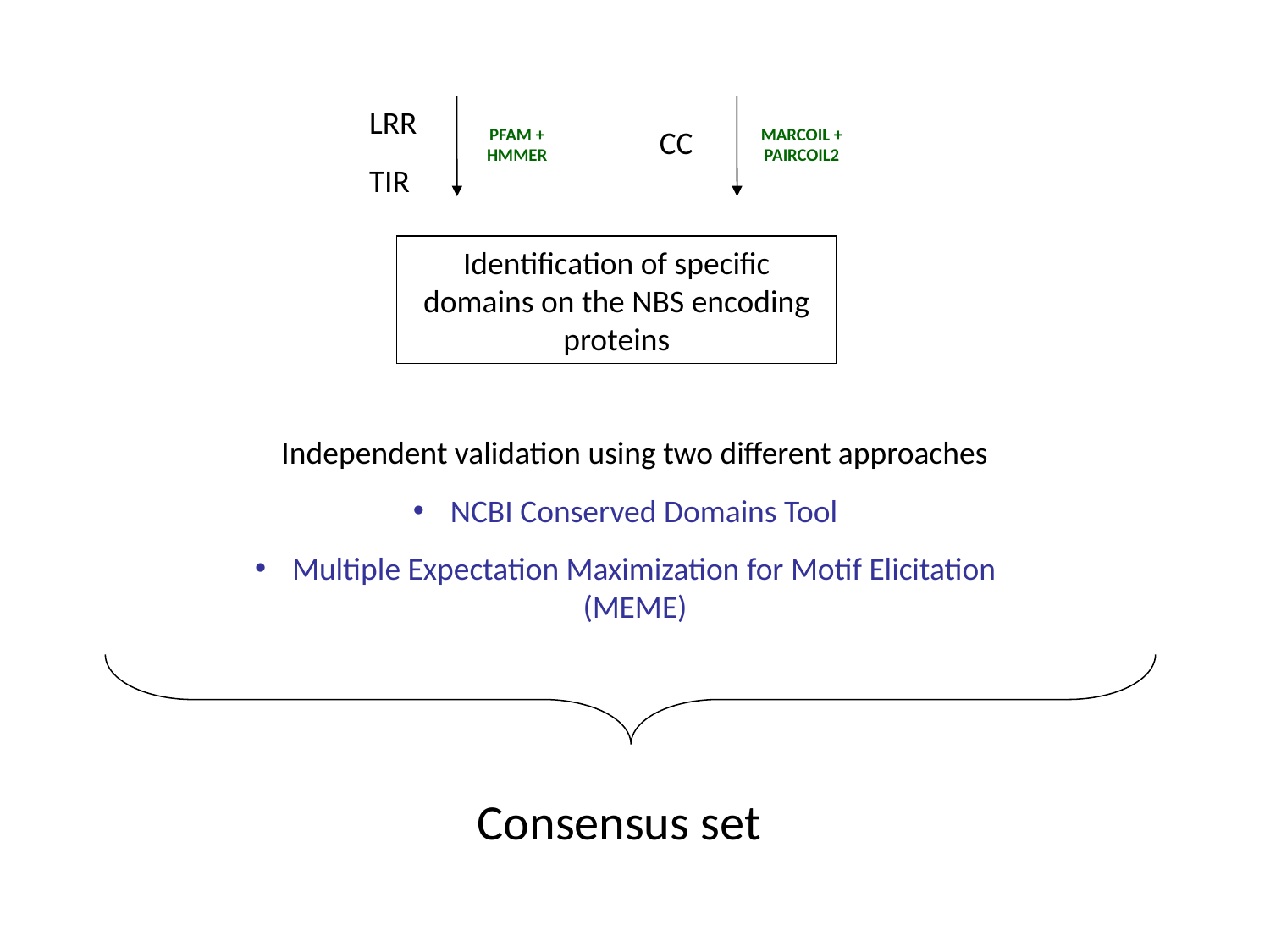

LRR
TIR
PFAM + HMMER
CC
MARCOIL + PAIRCOIL2
Identification of specific domains on the NBS encoding proteins
Independent validation using two different approaches
 NCBI Conserved Domains Tool
 Multiple Expectation Maximization for Motif Elicitation (MEME)
Consensus set
