# Supplementary figures and images for: Genome-Wide Identification and Mapping of NBS-Encoding Resistance Genes in Solanum tuberosum Group Phureja
Source: PLoS One. 2012 Apr 6;7(4):e34775. doi: 10.1371/journal.pone.0034775 (PMC3321028; doi:10.1371/journal.pone.0034775)

## Slide 1
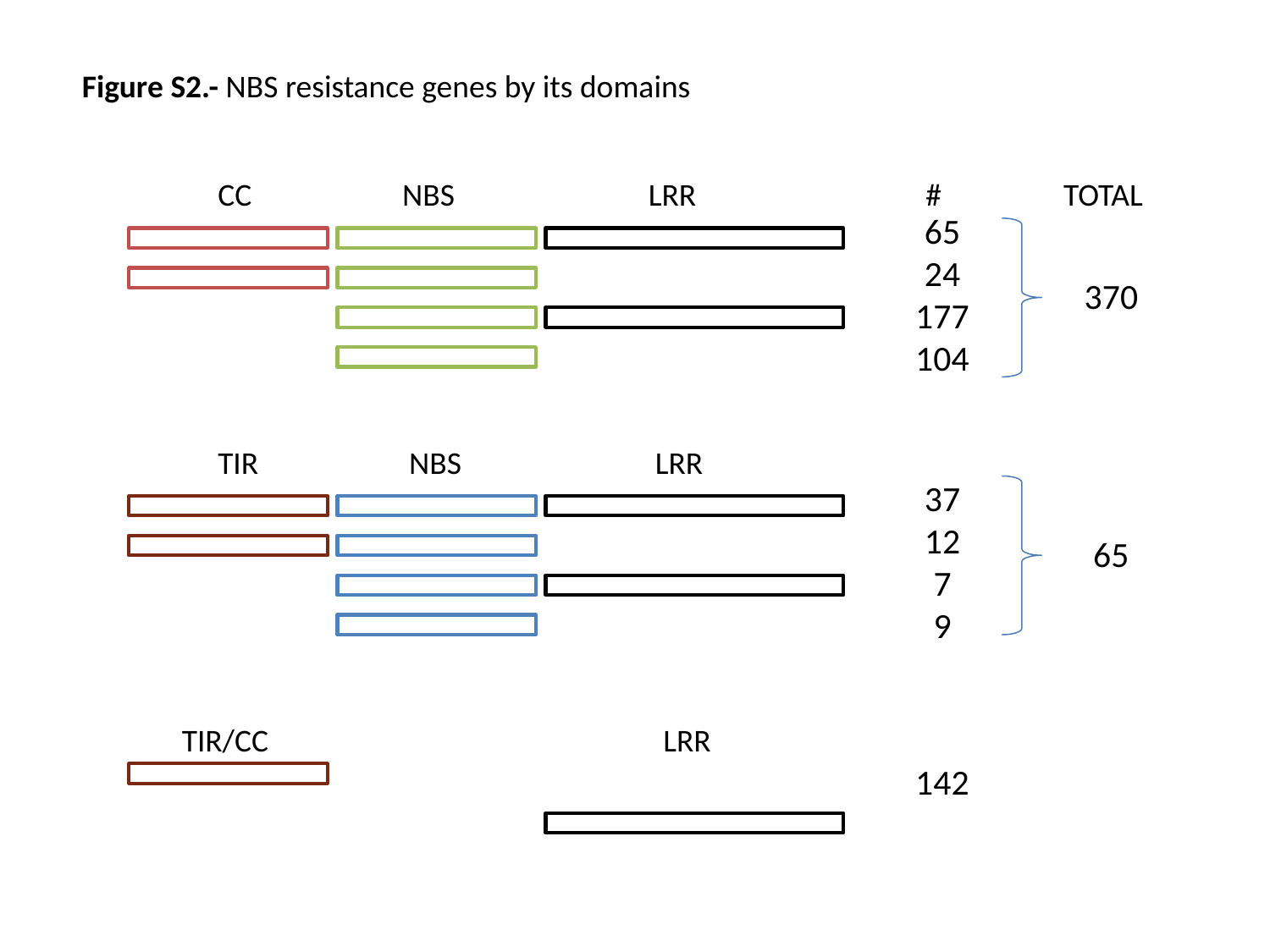

Figure S2.- NBS resistance genes by its domains
 CC NBS LRR # TOTAL
65
24
177
104
370
 TIR NBS LRR
37
12
7
9
65
 TIR/CC LRR
142

Supplement: Figure S2 — NBS resistance genes by its domains. (PPTX) [file pone.0034775.s002.pptx]

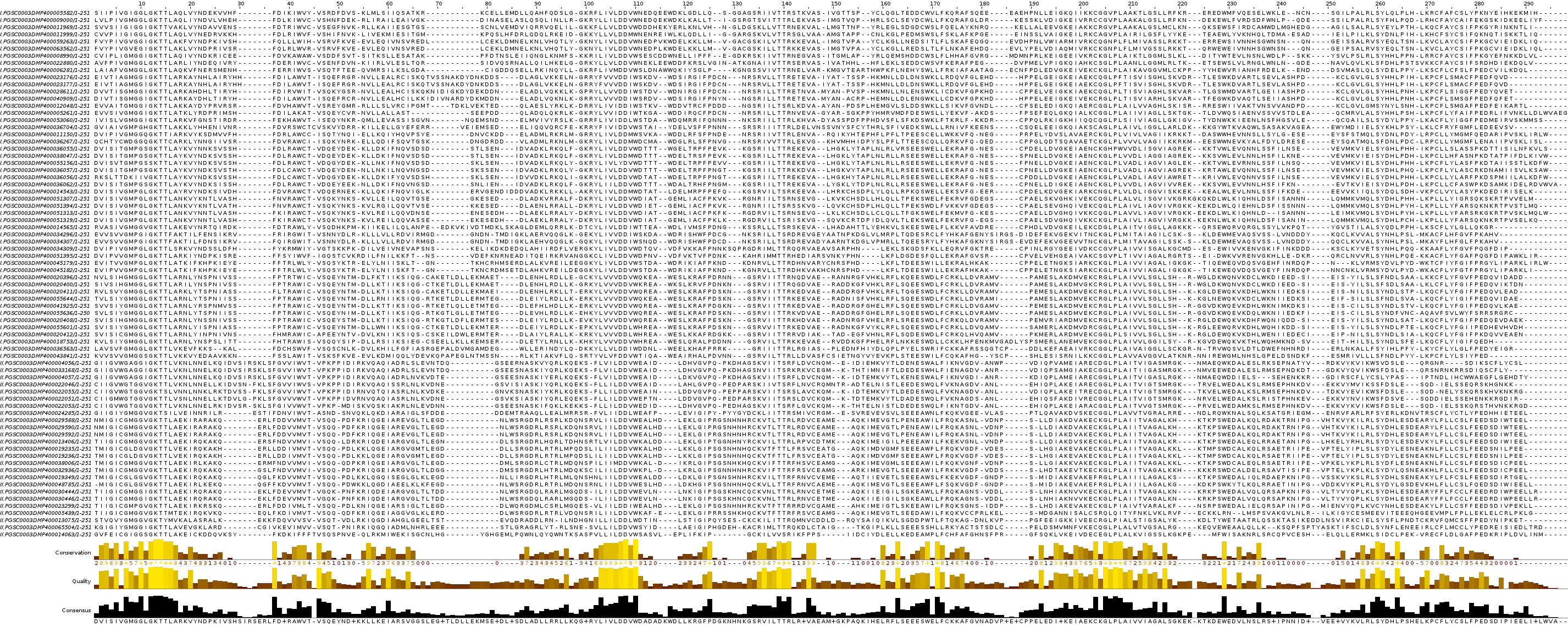

Supplement: Figure S4 — Alignment between CC(I) clade proteins vs. CC(II) clade proteins visualized using JALVIEW. (PNG) [file pone.0034775.s004.png]
