# Supplementary material for: Genome-Wide Identification and Mapping of NBS-Encoding Resistance Genes in Solanum tuberosum Group Phureja
Source: PLoS One. 2012 Apr 6;7(4):e34775. doi: 10.1371/journal.pone.0034775 (PMC3321028; doi:10.1371/journal.pone.0034775)
Supplement: Figure S3 — Phylogenetic tree including 224 NBS resistances genes found in this study plus reference R genes cloned from different species (in red). (PPT) [file pone.0034775.s003.ppt]

## Slide 1
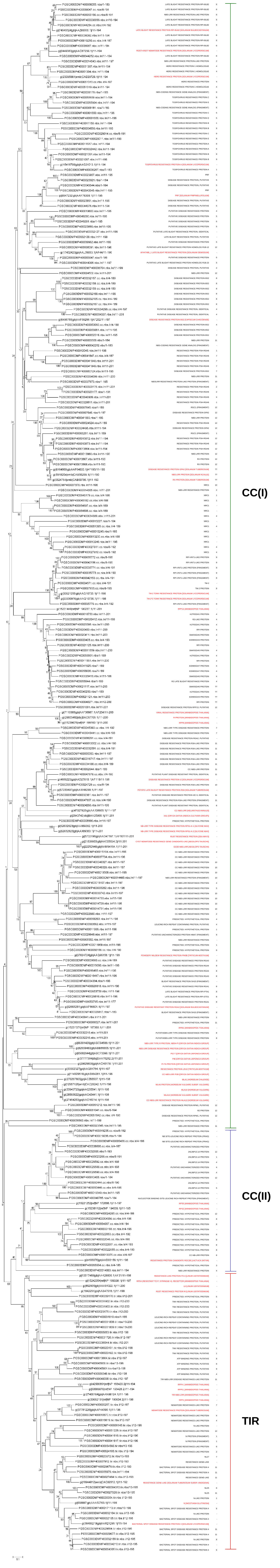

| LATE BLIGHT RESISTANCE PROTEIN RPI-BLB2 | 6 |
| --- | --- |
| LATE BLIGHT RESISTANCE PROTEIN RPI-BLB2 | 6 |
| NBS-LRR RESISTANCE PROTEIN-LIKE PROTEIN | 6 |
| LATE BLIGHT RESISTANCE PROTEIN RPI-BLB2 | 6 |
| LATE BLIGHT RESISTANCE PROTEIN RPI-BLB2 | 6 |
| LATE BLIGHT RESISTANCE PROTEIN RPI-BLB2 [SOLANUM BULBOCASTANUM] | - |
| LATE BLIGHT RESISTANCE PROTEIN RPI-BLB2 | 6 |
| LATE BLIGHT RESISTANCE PROTEIN RPI-BLB2 | 6 |
| LATE BLIGHT RESISTANCE PROTEIN RPI-BLB2 | 6 |
| ROOT-KNOT NEMATODE RESISTANCE PROTEIN [SOLANUM LYCOPERSICUM] | - |
| LATE BLIGHT RESISTANCE PROTEIN RPI-BLB2 | 6 |
| NBS-LRR RESISTANCE PROTEIN-LIKE PROTEIN | 2 |
| HERO RESISTANCE PROTEIN 1 HOMOLOGUE | 4 |
| HERO RESISTANCE PROTEIN 1 HOMOLOGUE | 4 |
| HERO RESISTANCE PROTEIN [SOLANUM LYCOPERSICUM] | - |
| HERO RESISTANCE PROTEIN | 4 |
| HERO RESISTANCE PROTEIN 1 HOMOLOGUE | 4 |
| NBS-CODING RESISTANCE GENE ANALOG (FRAGMENT) | 6 |
| TOSPOVIRUS RESISTANCE PROTEIN C | 6 |
| TOSPOVIRUS RESISTANCE PROTEIN E | 6 |
| NBS-CODING RESISTANCE GENE ANALOG (FRAGMENT) | 6 |
| TOSPOVIRUS RESISTANCE PROTEIN C | 5 |
| TOSPOVIRUS RESISTANCE PROTEIN D | 5 |
| TOSPOVIRUS RESISTANCE PROTEIN B | 1 |
| TOSPOVIRUS RESISTANCE PROTEIN B | 9 |
| TOSPOVIRUS RESISTANCE PROTEIN C | 9 |
| TOSPOVIRUS RESISTANCE PROTEIN E | 9 |
| TOSPOVIRUS RESISTANCE PROTEIN A | 9 |
| TOSPOVIRUS RESISTANCE PROTEIN A | 5 |
| TOSPOVIRUS RESISTANCE PROTEIN B | 9 |
| TOSPOVIRUS RESISTANCE PROTEIN B | 9 |
| TOSPOVIRUS RESISTANCE PROTEIN A [SOLANUM LYCOPERSICUM] | - |
| TOSPOVIRUS RESISTANCE PROTEIN A | 1 |
| PRF | 5 |
| DISEASE RESISTANCE PROTEIN, PUTATIVE | 5 |
| DISEASE RESISTANCE PROTEIN, PUTATIVE | 5 |
| PRF | 5 |
| PRF [SOLANUM PIMPINELLIFOLIUM] | - |
| DISEASE RESISTANCE PROTEIN, PUTATIVE | 5 |
| DISEASE RESISTANCE PROTEIN, PUTATIVE | 5 |
| PUTATIVE DISEASE RESISTANCE PROTEIN | 4 |
| PUTATIVE DISEASE RESISTANCE PROTEIN | 5 |
| PUTATIVE DISEASE RESISTANCE PROTEIN | 5 |
| PUTATIVE DISEASE RESISTANCE PROTEIN | 5 |
| PUTATIVE LATE BLIGHT RESISTANCE PROTEIN, IDENTICAL | - |
| LATE BLIGHT RESISTANCE PROTEIN, PUTATIVE | - |
| PUTATIVE DISEASE RESISTANCE PROTEIN | 5 |
| PUTATIVE LATE BLIGHT RESISTANCE PROTEIN HOMOLOG R1B-12 | 12 |
| AF447489\_1 LATE BLIGHT RESISTANCE PROTEIN [SOLANUM DEMISSUM] | - |
| PUTATIVE DISEASE RESISTANCE PROTEIN | 5 |
| PUTATIVE LATE BLIGHT RESISTANCE PROTEIN HOMOLOG R1B-23 | 5 |
| DISEASE RESISTANCE PROTEIN, PUTATIVE | 2 |
| NBS-LRR PROTEIN | 12 |
| DISEASE RESISTANCE PROTEIN BS2 | 8 |
| DISEASE RESISTANCE PROTEIN BS2 | 8 |
| DISEASE RESISTANCE PROTEIN BS2 | 8 |
| DISEASE RESISTANCE PROTEIN BS2 | 8 |
| DISEASE RESISTANCE PROTEIN BS2 | 8 |
| DISEASE RESISTANCE PROTEIN BS2 | 8 |
| PUTATIVE DISEASE RESISTANCE PROTEIN, IDENTICAL | 4 |
| PUTATIVE DISEASE RESISTANCE PROTEIN, IDENTICAL | 4 |
| DISEASE RESISTANCE PROTEIN BS2 [CAPSICUM CHACOENSE] | - |
| DISEASE RESISTANCE PROTEIN BS2 | 4 |
| DISEASE RESISTANCE PROTEIN BS2 | - |
| DISEASE RESISTANCE PROTEIN BS2 | 7 |
| NBS-LRR PROTEIN | 11 |
| NBS-CODING RESISTANCE GENE ANALOG (FRAGMENT) | - |
| RESISTANCE PROTEIN PSH-RGH6 | 1 |
| RESISTANCE PROTEIN PSH-RGH6 | 7 |
| RESISTANCE PROTEIN PSH-RGH6 | 7 |
| RESISTANCE PROTEIN PSH-RGH6 | 7 |
| DISEASE RESISTANCE PROTEIN BS2 | 7 |
| NBS-LRR PROTEIN | 11 |
| NBS/LRR RESISTANCE PROTEIN-LIKE PROTEIN (FRAGMENT) | 10 |
| RESISTANCE PROTEIN PSH-RGH6 | 2 |
| RESISTANCE PROTEIN PSH-RGH6 | 2 |
| RESISTANCE PROTEIN PSH-RGH6 | 4 |
| RESISTANCE PROTEIN PSH-RGH6 | 1 |
| RGC1 (FRAGMENT) | 12 |
| DISEASE RESISTANCE PROTEIN GPA2 | 12 |
| NBS-LRR PROTEIN | 12 |
| RESISTANCE PROTEIN PSH-RGH6 | 12 |
| DISEASE RESISTANCE PROTEIN GPA2 | 12 |
| RGC1 (FRAGMENT) | 1 |
| RESISTANCE PROTEIN PSH-RGH6 | 12 |
| RESISTANCE PROTEIN PSH-RGH6 | 12 |
| RESISTANCE PROTEIN PSH-RGH6 | 12 |
| NBS-LRR PROTEIN | 12 |
| RX PROTEIN | 12 |
| RX PROTEIN | 12 |
| DISEASE RESISTANCE PROTEIN GPA2 [SOLANUM TUBEROSUM] | ?? |
| NBS-LRR PROTEIN [SOLANUM ACAULE] | ?? |
| RX PROTEIN [SOLANUM TUBEROSUM] | ?? |
| NRC1 | 10 |
| NBS-LRR RESISTANCE PROTEIN | 2 |
| NRC1 | 1 |
| NRC1 | 1 |
| NRC1 | - |
| NRC1 | - |
| NRC1 | 4 |
| NRC1 | - |
| NRC1 | 4 |
| NRC1 | - |
| NRC1 | - |
| NRC1 | - |
| NRC1 | 4 |
| NRC1 | 4 |
| RPI-VNT1-LIKE PROTEIN | 9 |
| RPI-VNT1-LIKE PROTEIN | 9 |
| RPI-VNT1-LIKE PROTEIN (FRAGMENT) | 9 |
| RPI-VNT1-LIKE PROTEIN (FRAGMENT) | 9 |
| RPI-VNT1-LIKE PROTEIN (FRAGMENT) | 9 |
| TM-2 | - |
| TM-2 PROTEIN | 9 |
| TM-2 TOMV RESISTANCE PROTEIN [SOLANUM LYCOPERSICUM] | - |
| TM-2^2 TOMV RESISTANCE PROTEIN [SOLANUM LYCOPERSICUM] | - |
| RPI-VNT1-LIKE PROTEIN (FRAGMENT) | 9 |
| RPP13 [ARABIDOPSIS THALIANA] | - |
| HJTR2GH1 PROTEIN | 4 |
| R2-LIKE PROTEIN | 4 |
| HJTR2GH1 PROTEIN | 4 |
| RPI PROTEIN | 4 |
| SNKR2GH5 PROTEIN | 4 |
| EDNR2GH4 PROTEIN | 4 |
| RPI PROTEIN | - |
| SNKR2GH5 PROTEIN | 4 |
| HJTR2GH1 PROTEIN | 4 |
| RPI PROTEIN | 4 |
| EDNR2GH7 PROTEIN | 4 |
| EDNR2GH7 PROTEIN | 4 |
| SNKR2GH5 PROTEIN | 4 |
| R2 LATE BLIGHT RESISTANCE PROTEIN | 4 |
| SNKR2GH5 PROTEIN | ?? |
| HJTR2GH1 PROTEIN | ?? |
| EDNR2GH3 PROTEIN | ?? |
| RPI PROTEIN | ?? |
| DISEASE RESISTANCE PROTEIN RPP13, PUTATIVE | 12 |
| VIRAL RESISTANCE PROTEIN [ARABIDOPSIS THALIANA] | - |
| R-PROTEIN [ARABIDOPSIS THALIANA] | - |
| RPP8 [ARABIDOPSIS THALIANA] | - |
| NBS-LRR TYPE DISEASE RESISTANCE PROTEIN | 7 |
| NBS-LRR TYPE DISEASE RESISTANCE PROTEIN | 7 |
| DISEASE RESISTANCE PROTEIN RGA2, PUTATIVE | - |
| DISEASE RESISTANCE PROTEIN R3A-LIKE PROTEIN | 11 |
| DISEASE RESISTANCE PROTEIN R3A-LIKE PROTEIN | 11 |
| DISEASE RESISTANCE PROTEIN R3A-LIKE PROTEIN | 11 |
| DISEASE RESISTANCE PROTEIN R3A-LIKE PROTEIN | 11 |
| DISEASE RESISTANCE PROTEIN R3A-LIKE PROTEIN | 11 |
| CC-NBS-LRR PROTEIN | 11 |
| PUTATIVE PLANT DISEASE RESISTANT PROTEIN, IDENTICAL | 11 |
| DISEASE RESISTANCE PROTEIN I2 [SOLANUM LYCOPERSICUM] | - |
| DISEASE RESISTANCE PROTEIN R3A-LIKE PROTEIN | 10 |
| POTATO LATE BLIGHT RESISTANCE PROTEIN R3A [SOLANUM TUBEROSUM] | - |
| PUTATIVE DISEASE RESISTANCE PROTEIN I2C-5, IDENTICAL | 11 |
| DISEASE RESISTANCE PROTEIN R3A-LIKE PROTEIN (FRAGMENT) | 11 |
| PUTATIVE PLANT DISEASE RESISTANT PROTEIN, IDENTICAL | 8 |
| CC-NBS-LRR [HELIANTHUS ANNUUS] | - |
| XA1 [ORYZA SATIVA (INDICA CULTIVAR-GROUP)] | - |
| PREDICTED: HYPOTHETICAL PROTEIN | 3 |
| NB-LRR TYPE DISEASE RESISTANCE PROTEIN RPS1-K-1 [GLYCINE MAX] | - |
| NB-LRR TYPE DISEASE RESISTANCE PROTEIN RPS1-K-2 [GLYCINE MAX] | - |
| RUST RESISTANCE PROTEIN [ZEA MAYS] | - |
| CYST NEMATODE RESISTANCE GENE CANDIDATE-LIKE [AEGILOPS TAUSCHII] | - |
| GO35 NBS-LRR [AEGILOPS TAUSCHII] | - |
| CC-NBS-LRR RESISTANCE PROTEIN | 4 |
| CC-NBS-LRR RESISTANCE PROTEIN | - |
| CC-NBS-LRR RESISTANCE PROTEIN | 10 |
| CC-NBS-LRR RESISTANCE PROTEIN | 10 |
| CC-NBS-LRR RESISTANCE PROTEIN | 10 |
| NBS-LRR RESISTANCE PROTEIN (FRAGMENT) | 10 |
| CC-NBS-LRR RESISTANCE PROTEIN | 4 |
| CC-NBS-LRR RESISTANCE PROTEIN | 5 |
| CC-NBS-LRR RESISTANCE PROTEIN | 10 |
| CC-NBS-LRR RESISTANCE PROTEIN | 10 |
| CC-NBS-LRR RESISTANCE PROTEIN | 10 |
| CC-NBS-LRR RESISTANCE PROTEIN | 10 |
| CC-NBS-LRR RESISTANCE PROTEIN | 2 |
| PREDICTED: HYPOTHETICAL PROTEIN | 4 |
| LEUCINE-RICH REPEAT CONTAINING PROTEIN, PUTATIVE | 4 |
| PREDICTED: HYPOTHETICAL PROTEIN | 1 |
| PUTATIVE UNCHARACTERIZED PROTEIN HMA7 (FRAGMENT) | 9 |
| NBS-LRR RESISTANCE PROTEIN | - |
| PREDICTED: HYPOTHETICAL PROTEIN | 1 |
| PREDICTED: HYPOTHETICAL PROTEIN | 9 |
| POWDERY MILDEW RESISTANCE PROTEIN PM3B [TRITICUM AESTIVUM] | - |
| PUTATIVE DISEASE RESISTANCE PROTEIN RGA3 | 3 |
| PUTATIVE DISEASE RESISTANCE PROTEIN RGA4 | 9 |
| PUTATIVE DISEASE RESISTANCE PROTEIN RGA4 | 8 |
| PUTATIVE DISEASE RESISTANCE PROTEIN RGA3 | 8 |
| BLIGHT RESISTANCE PROTEIN SH10 (FRAGMENT) | - |
| PUTATIVE DISEASE RESISTANCE PROTEIN RGA1 | 8 |
| LATE BLIGHT RESISTANCE PROTEIN RPI-PTA1 | 8 |
| LATE BLIGHT RESISTANCE PROTEIN RPI-STO1 | 8 |
| PUTATIVE DISEASE RESISTANCE PROTEIN RGA1 | 8 |
| PUTATIVE DISEASE RESISTANT PROTEIN RGA2 [SOLANUM BULBOCASTANUM] | - |
| BLIGHT RESISTANCE PROTEIN SH10 (FRAGMENT) | 8 |
| NBS-LRR RESISTANCE PROTEIN | 1 |
| PREDICTED: HYPOTHETICAL PROTEIN | 11 |
| RPM1 [ARABIDOPSIS THALIANA] | - |
| PUTATIVEBS-LRR TYPE DISEASE RESISTANCE PROTEIN | 8 |
| PUTATIVEBS-LRR TYPE DISEASE RESISTANCE PROTEIN | 8 |
| NBS-LRR TYPE R PROTEIN, NBS4-PI [ORYZA SATIVA INDICA GROUP] | - |
| NBS-LRR DISEASE RESISTANCE PROTEIN [ORYZA SATIVA INDICA GROUP] | - |
| PIZ-T [ORYZA SATIVA JAPONICA GROUP] | - |
| PIB [ORYZA SATIVA JAPONICA GROUP] | - |
| PI-TA PROTEIN [ORYZA SATIVA JAPONICA GROUP] | - |
| RESISTANCE PROTEIN LR10 [TRITICUM AESTIVUM] | - |
| CC-NBS-LRR PI36 [ORYZA SATIVA INDICA GROUP] | - |
| MLA1 [HORDEUM CHILENSE] | - |
| MLA6 PROTEIN [HORDEUM VULGARE SUBSP. VULGARE] | - |
| MLA10 [HORDEUM VULGARE] | - |
| MLA12 [HORDEUM VULGARE SUBSP. VULGARE] | - |
| CC-NBS-LRR RESISTANCE PROTEIN MLA13 [HORDEUM VULGARE] | - |
| DISEASE RESISTANCE PROTEIN RPM1, PUTATIVE | 12 |
| R2 PROTEIN | 12 |
| DISEASE RESISTANCE PROTEIN RPM1, PUTATIVE | 12 |
| PREDICTED: HYPOTHETICAL PROTEIN | - |
| NBS-LRR RESISTANCE PROTEIN | 11 |
| PREDICTED: HYPOTHETICAL PROTEIN | - |
| NB SITE-LEUCINE RICH REPEAT PROTEIN (FRAG) | - |
| NB SITE-LEUCINE RICH REPEAT PROTEIN (FRAG) | - |
| PUTATIVE UNCHARACTERIZED PROTEIN | - |
| JHL06P13.14 PROTEIN | 12 |
| JHL06P13.14 PROTEIN | 1 |
| JHL06P13.14 PROTEIN | 4 |
| PUTATIVE UNCHARACTERIZED PROTEIN | 4 |
| JHL06P13.14 PROTEIN | 4 |
| PUTATIVE UNCHARACTERIZED PROTEIN | 12 |
| JHL06P13.14 PROTEIN | - |
| JHL06P13.14 PROTEIN | 5 |
| PUTATIVE UNCHARACTERIZED PROTEIN | - |
| NUCLEOTIDE BINDING SITE-LEUCINE RICH REPEAT PROTEIN (FRAGMENT) | - |
| RPS5 [ARABIDOPSIS THALIANA] | - |
| RPS2 [ARABIDOPSIS THALIANA] | - |
| PREDICTED: HYPOTHETICAL PROTEIN | 12 |
| PREDICTED: HYPOTHETICAL PROTEIN | 8 |
| PREDICTED: HYPOTHETICAL PROTEIN | 8 |
| PREDICTED: HYPOTHETICAL PROTEIN | - |
| PREDICTED: HYPOTHETICAL PROTEIN | 8 |
| PREDICTED: HYPOTHETICAL PROTEIN | 8 |
| PREDICTED: HYPOTHETICAL PROTEIN | 8 |
| PREDICTED: HYPOTHETICAL PROTEIN | 8 |
| CC-NB-LRR PROTEIN | 8 |
| RESISTANCE PROTEIN CANDIDATE RGC2B [LACTUCA SATIVA] | - |
| PREDICTED: HYPOTHETICAL PROTEIN | 3 |
| NBS-LRR RESISTANCE PROTEIN | 4 |
| RESISTANCE-LIKE PROTEIN P2-A [LINUM USITATISSIMUM] | - |
| RPS4 (RESISTANT TO P. SYRINGAE 4); RECEPTOR [ARABIDOPSIS THALIANA] | - |
| L6 [LINUM USITATISSIMUM] | - |
| RUST RESISTANCE PROTEIN M [LINUM USITATISSIMUM] | - |
| PREDICTED: HYPOTHETICAL PROTEIN | 5 |
| TMV RESISTANCE PROTEIN, PUTATIVE | 1 |
| TMV RESISTANCE PROTEIN, PUTATIVE | 1 |
| TMV RESISTANCE PROTEIN, PUTATIVE | 2 |
| LEUCINE-RICH REPEAT-CONTAINING PROTEIN, PUTATIVE | 1 |
| LEUCINE-RICH REPEAT-CONTAINING PROTEIN, PUTATIVE | 1 |
| LEUCINE-RICH REPEAT-CONTAINING PROTEIN, PUTATIVE | 1 |
| TMV RESISTANCE PROTEIN, PUTATIVE | - |
| LEUCINE-RICH REPEAT-CONTAINING PROTEIN, PUTATIVE | 1 |
| LEUCINE-RICH REPEAT-CONTAINING PROTEIN, PUTATIVE | 1 |
| TMV RESISTANCE PROTEIN, PUTATIVE | 1 |
| TMV RESISTANCE PROTEIN, PUTATIVE | 1 |
| ATP BINDING PROTEIN, PUTATIVE | 9 |
| ATP BINDING PROTEIN, PUTATIVE | 9 |
| ATP BINDING PROTEIN, PUTATIVE | 9 |
| ATP BINDING PROTEIN, PUTATIVE | 4 |
| TIR-NBS-LRR DISEASE RESISTANCE-LIKE PROTEIN | 11 |
| RPP4 ( [ARABIDOPSIS THALIANA] | - |
| RPP5 ( [ARABIDOPSIS THALIANA] | - |
| TIR-NBS-LRR [ARABIDOPSIS THALIANA] | - |
| RPP1 [ARABIDOPSIS THALIANA] | - |
| NEMATODE RESISTANCE-LIKE PROTEIN | 7 |
| NEMATODE RESISTANCE PROTEIN [SOLANUM TUBEROSUM] | - |
| NEMATODE RESISTANCE-LIKE PROTEIN | 4 |
| NEMATODE RESISTANCE-LIKE PROTEIN | 4 |
| N-LIKE PROTEIN | - |
| NEMATODE RESISTANCE-LIKE PROTEIN | 12 |
| N PROTEIN (FRAGMENT) | 1 |
| N PROTEIN (FRAGMENT) | 1 |
| NEMATODE RESISTANCE-LIKE PROTEIN | 6 |
| NEMATODE RESISTANCE-LIKE PROTEIN | 2 |
| NL27 | 7 |
| RESISTANCE GENE-LIKE | - |
| BACTERIAL SPOT DISEASE RESISTANCE PROTEIN 4 | 11 |
| BACTERIAL SPOT DISEASE RESISTANCE PROTEIN 4 | 11 |
| RESISTANCE GENE-LIKE | 11 |
| RESISTANCE GENE-LIKE [SOLANUM TUBEROSUM SUBSP. ANDIGENA] | - |
| NL25 | 6 |
| NL25 | 11 |
| N-LIKE PROTEIN | 1 |
| N [NICOTIANA GLUTINOSA] | - |
| BACTERIAL SPOT DISEASE RESISTANCE PROTEIN 4 | 6 |
| N-LIKE PROTEIN | - |
| BACTERIAL SPOT DISEASE RESISTANCE PROTEIN 4 | - |
| BACTERIAL SPOT DISEASE RESISTANCE PROTEIN 4 [SOLANUM LYCOPERSICUM] | - |
| BACTERIAL SPOT DISEASE RESISTANCE PROTEIN 4 | 11 |
| BACTERIAL SPOT DISEASE RESISTANCE PROTEIN 4 | 11 |
| N-LIKE PROTEIN | 8 |
| BACTERIAL SPOT DISEASE RESISTANCE PROTEIN 4 | - |
| BACTERIAL SPOT DISEASE RESISTANCE PROTEIN 4 | 6 |
CC(I)
CC(II)
TIR
