# Supplementary material for: Genome-Wide Identification and Mapping of NBS-Encoding Resistance Genes in Solanum tuberosum Group Phureja
Source: PLoS One. 2012 Apr 6;7(4):e34775. doi: 10.1371/journal.pone.0034775 (PMC3321028; doi:10.1371/journal.pone.0034775)
Supplement: Figure S5 — Differences between the domains of CC(I) and CC(II) proteins visualized with MEME. (PPTX) [file pone.0034775.s005.pptx]

## Slide 1
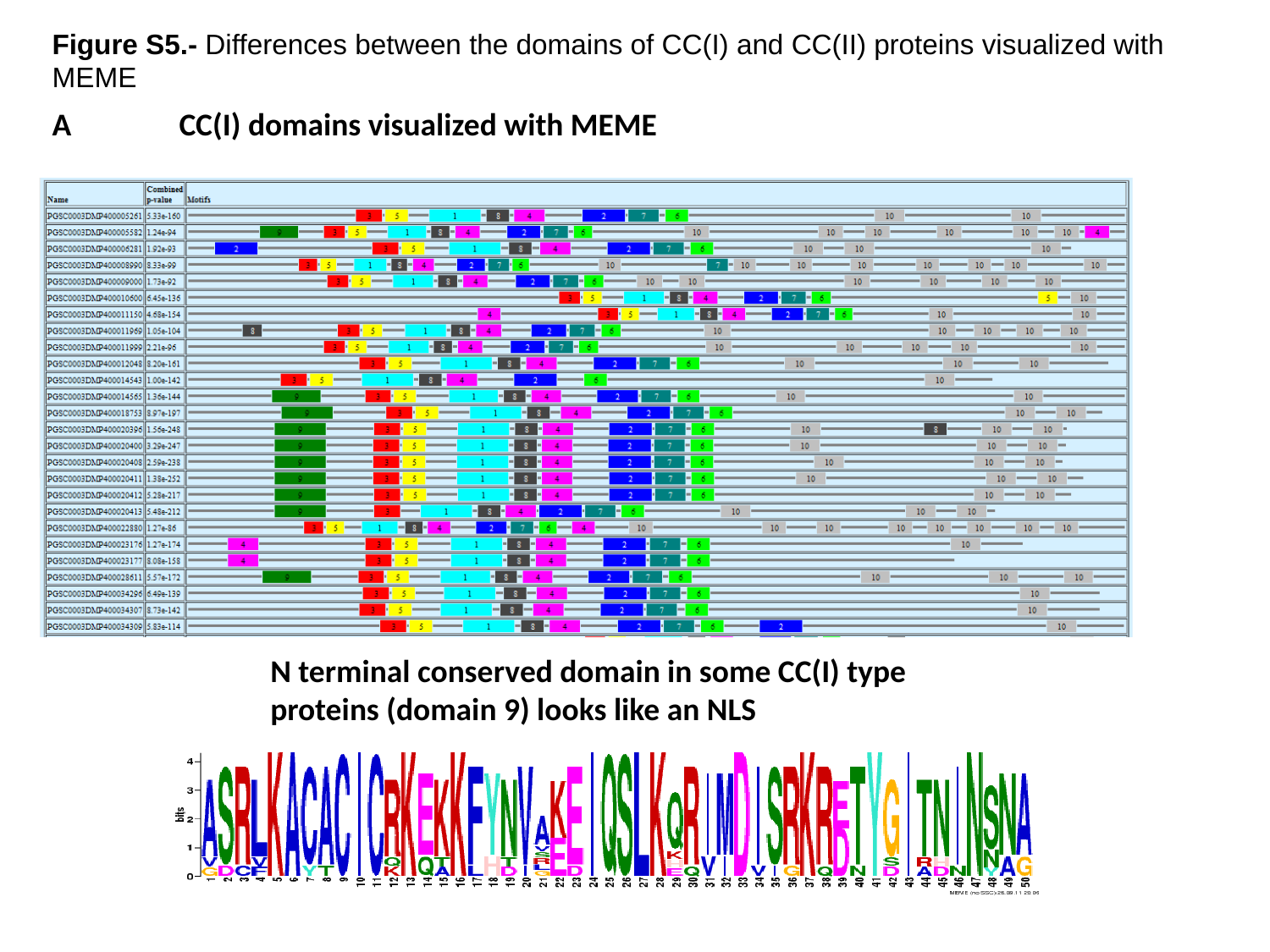

Figure S5.- Differences between the domains of CC(I) and CC(II) proteins visualized with MEME
A	CC(I) domains visualized with MEME
N terminal conserved domain in some CC(I) type proteins (domain 9) looks like an NLS

## Slide 2
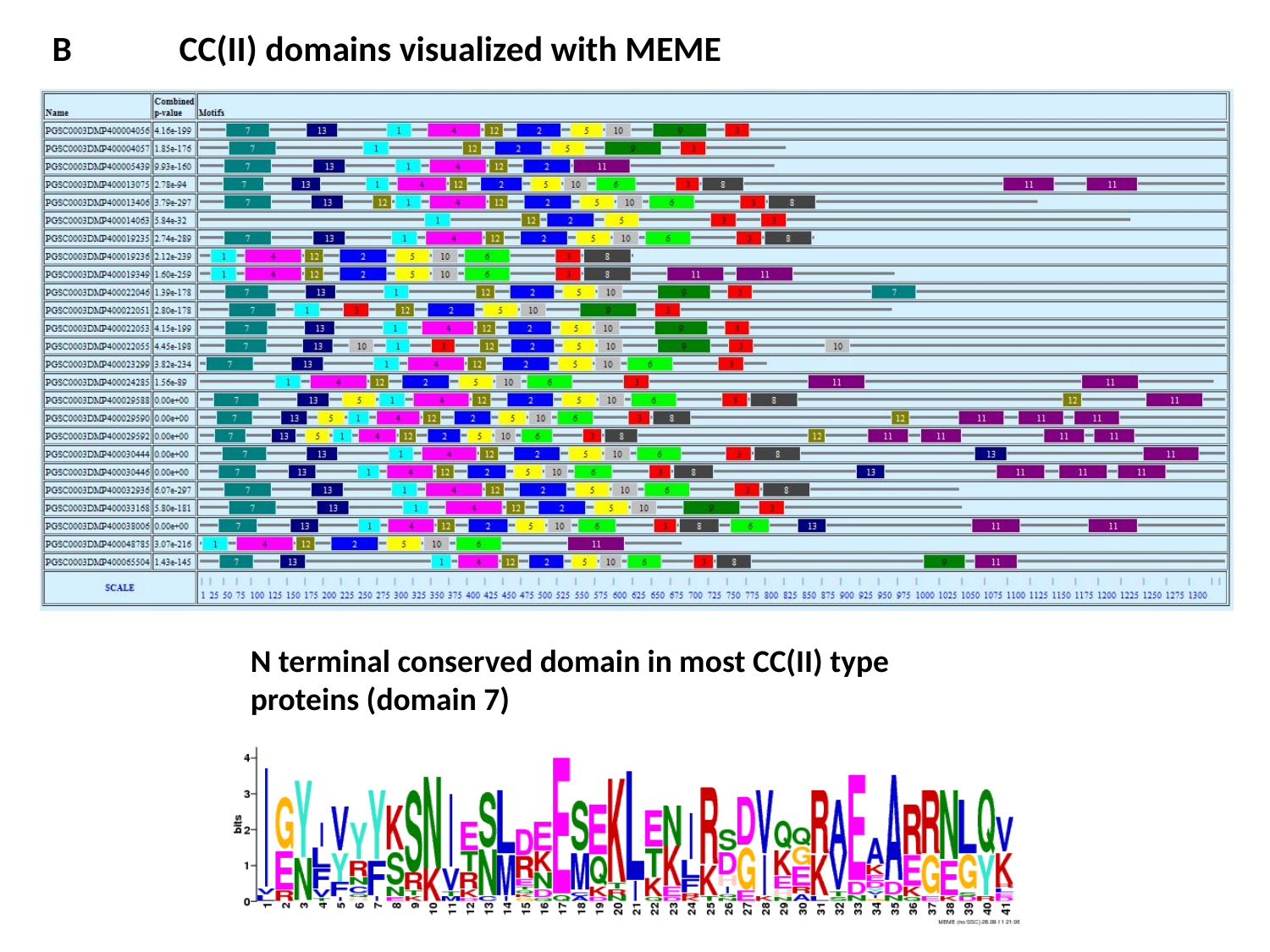

B	CC(II) domains visualized with MEME
N terminal conserved domain in most CC(II) type proteins (domain 7)
